# Human cellular CYBA UTR sequences increase mRNA translation without affecting the half-life of recombinant RNA transcripts

**DOI:** 10.1038/srep39149

**Published:** 2016-12-15

**Authors:** Mehrije Ferizi, Manish K. Aneja, Elizabeth R. Balmayor, Zohreh Sadat Badieyan, Olga Mykhaylyk, Carsten Rudolph, Christian Plank

**Affiliations:** 1 Institute of Molecular Immunology- Experimental Oncology, Klinikum rechts der Isar, Technische Universität München, Munich, 81675, Germany; 2 Ethris GmbH, Planegg, 82152, Germany; 3 Experimental Trauma Surgery, Klinikum rechts der Isar, Technische Universität München, Munich, 81675, Germany

## Abstract

Modified nucleotide chemistries that increase the half-life (T_1/2_) of transfected recombinant mRNA and the use of non-native 5′- and 3′-untranslated region (UTR) sequences that enhance protein translation are advancing the prospects of transcript therapy. To this end, a set of UTR sequences that are present in mRNAs with long cellular T_1/2_ were synthesized and cloned as five different recombinant sequence set combinations as upstream 5′-UTR and/or downstream 3′-UTR regions flanking a reporter gene. Initial screening in two different cell systems *in vitro* revealed that cytochrome b-245 alpha chain (CYBA) combinations performed the best among all other UTR combinations and were characterized in detail. The presence or absence of CYBA UTRs had no impact on the mRNA stability of transfected mRNAs, but appeared to enhance the productivity of transfected transcripts based on the measurement of mRNA and protein levels in cells. When CYBA UTRs were fused to human bone morphogenetic protein 2 (hBMP2) coding sequence, the recombinant mRNA transcripts upon transfection produced higher levels of protein as compared to control transcripts. Moreover, transfection of human adipose mesenchymal stem cells with recombinant hBMP2-CYBA UTR transcripts induced bone differentiation demonstrating the osteogenic and therapeutic potential for transcript therapy based on hybrid UTR designs.

The direct delivery of recombinant RNA transcripts to the cytoplasm, results in the immediate translation of its encoded therapeutic protein. The delivery of a bolus of exogenous mRNA is intended to have a major transient impact on protein production and turnover without directly altering or relying on transcriptional activation of the corresponding endogenous gene^1^,^2^,^3^. Control elements present in individual mRNA sequences including the 5′-UTR, 3′-UTR as well as sequence elements within the coding sequence can all impact protein translation and mRNA stability. In particular, UTRs are known to play a pivotal role in protein translation and mRNA stability through the interaction with RNA-binding proteins (RBPs)^1^,^4^.

Another cis-acting element located in the mRNA with major impact on stability is the poly(A) tail. Poly(A) tails serve to stabilize RNA transcripts^5^,^6^,^7^ by protecting and delaying 3′-end degradation. RNA can be degraded by 3 major pathways which have been described elaborately in other studies^8^,^9^.

Therefore, nascent mRNAs have highly variable mRNA half-lives ranging from several minutes to hours. For instance β-globin mRNA is extraordinary stable with a T_1/2_ of 16 to 48 h, whereas transcripts from hexokinase II are quite short-lived ~3.6 h^10^,^11^. In general, transcripts with a relatively long T_1/2_ are involved in aspects of cellular metabolism whereas shorter-lived transcripts often encode genes involved in e.g. transcriptional regulation^12^,^13^,^14^.

Collectively, the range of different control elements present in RNA sequences can impact their secondary and tertiary structures and represent an important realm of features to evaluate in the development of effective transcript therapies. Until now the delivery of exogenous mRNA has primarily been focused on the development of immunotherapeutic vaccines^15^,^16^. In contrast, the notion of transcript therapy for the delivery aims to alter protein translation within a cell to gain a therapeutic or preventive effect. Therefore, several approaches have been implemented to stabilize mRNA and increase translation.

Among these is the sequence optimization of mRNA, extension of the poly(A) tail to 150 nucleotides or longer, introduction of non-native UTRs and the inclusion of chemically-modified nucleotides during *in vitro* transcription^17^,^18^,^19^. Holtkamp *et al*. demonstrated that a poly(A) length of 120 nt enhances reporter protein translation. Furthermore, they could show that insertion of one copy of the 3′-UTR from human β-globin serves to increase protein translation which was further increased when two copies of the same 3′-UTR were introduced back-to-back^18^. In another study by Kormann and colleagues, translation was maximized and innate immune response was reduced in a mouse lung disease model by introducing chemically-modified nucleotides during *in vitro* transcription of mRNA (IVT-mRNA), coding for surfactant protein B (SP-B) with a 25% replacement of cytidine and uridine by modified 5-methyl cytidine (5-methyl CTP) and 2-thio uridine (2-thio UTP), respectively^19^. Other studies have reported that modified nucleotide(s) within the mRNA structure have a strong impact on protein translation and enable reprogramming of human cells to pluripotency, vascular regeneration, as well as bone regeneration *in vitro* and *in vivo*^20^,^21^,^22^,^23^. In the latter example, modified mRNA coding for human bone morphogenetic protein 2 (hBMP2) was used to induce bone regeneration^22^,^23^. Recently, we demonstrated that the usage of chemically-modified mRNA (cmRNA) consisting of 5-methyl CTP and 2-thio UTP, a long poly(A) and no UTRs resulted in osteogenic differentiation *in vitro* in mesenchymal stem cells showing significantly elevated expression of osteogenesis related genes such RunX2, a transcription factor, and osteopontin (OPN), an extracellular protein^22^. Further *ex vivo* and *in vivo* experiments, in human adipose tissue and long bone defects in rats, respectively, have confirmed transcript therapy as a potential therapeutic strategy^22^.

One objective of the current study was to combine UTR based mRNA design with the use of modified nucleotides aimed at improving the translational efficiency and stability of mRNA-encoding hBMP2. In one approach, a set of UTR sequences were identified in natural long-lived mRNAs^24^,^25^ and then explored to assess whether any of these could be categorized as possessing inherent stability or translation enhancing properties. We also explored whether long-lived UTRs in combination with chemical modification of mRNA and a poly(A) tail would be synergistic with respect to persistence of protein translation as compared to mRNAs lacking such UTRs. Based on mRNA stability data, a set of UTRs was selected and cloned upstream or downstream flanking the reporter coding sequence.

Specifically, UTRs from human cytochrome b-245 alpha polypeptide (CYBA), 2-4-dienoyl-CoA reductase (DECR1), glia maturation factor, gamma (GMFG), mitogen-activated protein-binding protein-interacting protein (MAPBPIP) and myosin, light chain 6B (MYL6B) were chosen^24^,^25^. Five different combinations were investigated for each UTR: 5′-UTR, 3′-UTR, 5′-+3′-UTR (5 + 3), 5′-+2x3′UTR (5 + 2X3) and two copies of 3′-UTR (2X3). Quantitative real time PCR (qRT-PCR) was used to determine the physical T_1/2_ in two different cell lines. As highest protein amounts were observed for CYBA UTR combinations, additional experiments in C2C12 cells and in human adipose mesenchymal stem cells (hAMSCs) were conducted with constructs carrying hBMP2 coding sequence flanked by CYBA UTRs. Reporter protein amounts and recombinant mRNA quantity from each of the compared transcripts were assessed using bioluminescence and qRT-PCR measurement, respectively. The functionality of the recombinant hBMP2-CYBA mRNA constructs was verified by inducing osteogenic differentiation in hAMSCs *in vitro*.

## Results

**Figure 1:**
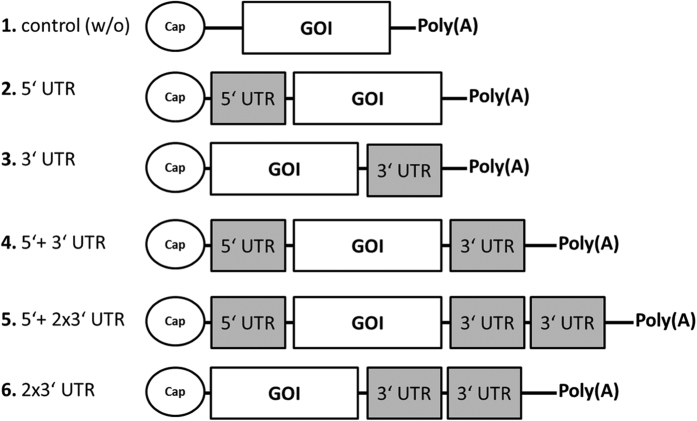
Schematic representation of all combinations for one cellular UTR. Cellular UTRs (grey) were inserted upstream and/or downstream of the gene of interest (GOI). All produced mRNAs were synthesized using chemically modified nucleotides in the IVT mix and were ARCA capped (circle).

UTRs from five mRNAs, namely, CYBA, DECR1, GMFG, MAPBPIP and MYL6B, reported to have long half-lives with high levels of expression, were selected for analysis with reporter genes. To generate recombinant RNA transcripts with low immunogenicity, all sequences were cloned in a plasmid vector that would serve as a template for run-off transcription reactions containing a 5′ T7 promoter, cloning sites, and a poly(A) tail. Modified nucleotides were used during the generation of recombinant transcripts by *in vitro* RNA synthesis according to Kormann *et al*.^19^. The selected UTRs were cloned upstream and/or downstream of the gene of interest (GOI), respectively. UTR sequences were obtained from the UTR database (http://utrdb.ba.itb.cnr.it/). For each UTR, five different sequence combinations were designed comprising: 5′- and 3′- UTR alone, 5′- +3′-UTR (5 + 3), 5′- +2x3′UTR (5 + 2X3) and two copies of 3′-UTR (2X3) (Fig. 1). The integrity of the recombinant chemically-modified transcripts carrying *Metridia* Luciferase (*Met*Luc) was confirmed by agarose gel (1%) electrophoresis (Supplementary Fig. S1). The sizes (bases) of all *Met*Luc transcripts are listed in Supplementary Table S1. All chemically modified transcripts comprised a single homogeneous band of expected size by electrophoresis suggesting purity without degradation of mRNA product.

### Screening of different UTRs in NIH3T3 and A549 cells: Area under the curve

**Figure 2:**
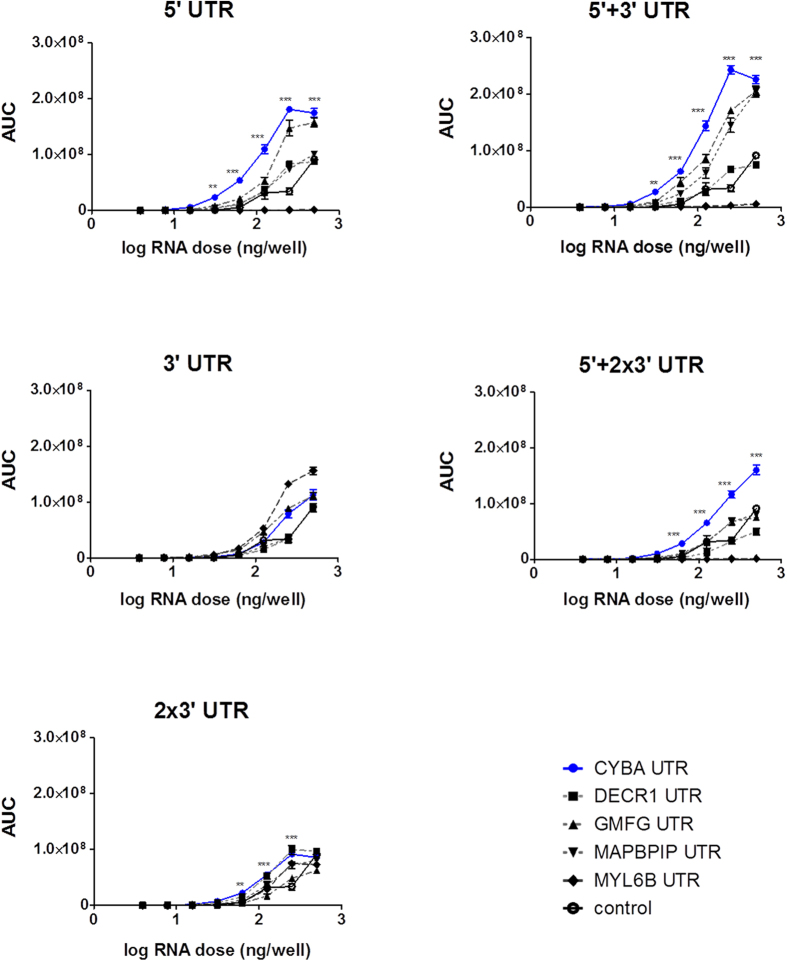
Mean total protein production over time (Area Under the Curve - AUC) in NIH3T3 cells. Reporter protein was measured over 5-days resulting in a total protein amount. AUC values were plotted for each cellular UTR against the dose (log scale). Statistical significance was assessed by 2-way ANOVA test with p values: **p < 0.01, ***p < 0.001.

**Figure 3:**
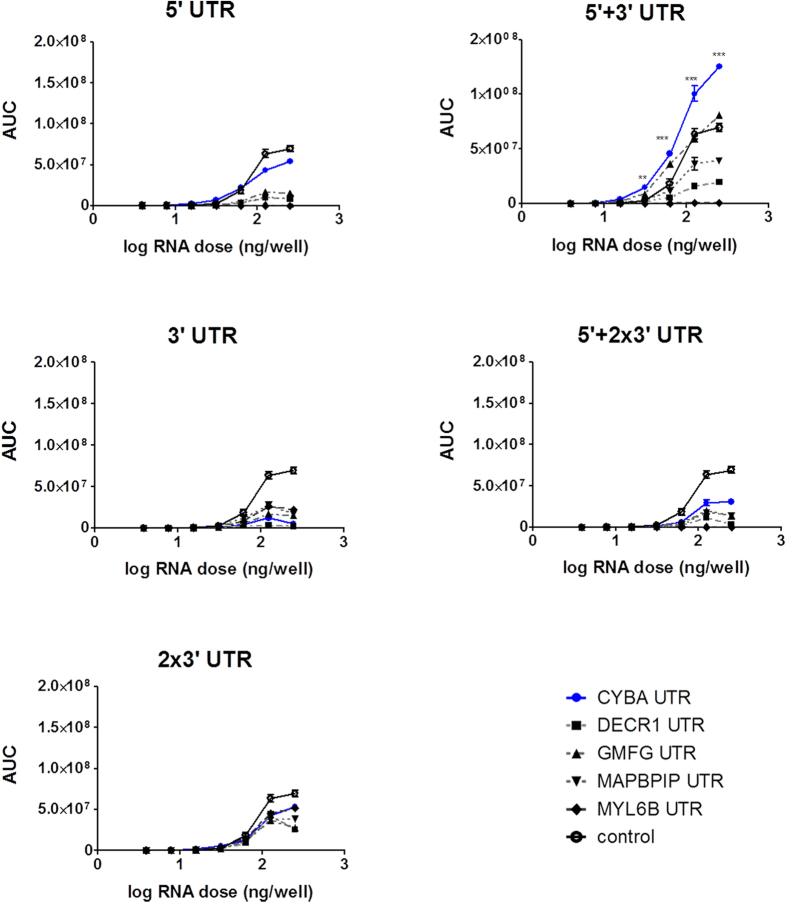
Mean total protein production over time (Area Under the Curve - AUC) in A549 cells. Reporter protein was measured over 5-days resulting in a total protein amount. AUC values were plotted for each cellular UTR against the dose (log scale). Statistical significance was assessed by 2-way ANOVA test with p values: **p < 0.01, ***p < 0.001.

In a preliminary experiment, different transfection reagents (TfR) were compared for mRNA delivery in NIH3T3 and A549 cell lines (Supplementary Fig. S2). The best TfR, Dreamfect Gold (DFG), with respect to translation efficiency and cell viability, was selected for all further transfection studies. To assess the impact of various UTRs on translation, 25 mRNA constructs, furnished with various cellular UTRs (Fig. 1) were screened in NIH3T3 and A549 cell lines. All mRNA constructs coded for *Metridia* luciferase (*Met*Luc), which is secreted into the medium. Dose-dependent translation kinetics were analyzed for up to 5 days post-transfection and total protein translation, also defined as area under the curve (AUC), was used to compare the different UTR constructs (Figs 2 and 3). In murine NIH3T3 cells, mRNA doses ranging from 3.9 ng/well to 500 ng/well and in human A549 cells mRNA doses of 3.9 ng/well up to 250 ng/well were transfected. In NIH3T3 cells, UTRs from CYBA gene, in all tested combinations, with the exception of 3′-UTR alone, resulted in significantly higher protein level compared to the control *Met*Luc mRNA without UTRs (Fig. 2). This was observed over a broad range of transfected mRNA doses. Though the effects were not so strong in human A549 cells, significantly higher translation was observed with *Met*Luc-CYBA 5 + 3 recombinant mRNA (Fig. 3).

In summary, it could be shown that incorporation of CYBA UTRs into a mRNA sequence significantly increased protein levels post-transfection. Since *Met*Luc is also a secreted protein like our target protein (hBMP2), these data in our opinion are more comparable than they would be, had we used EGFP or other intracellular luciferases as a marker. Moreover, *Metridia* luciferase activity can be measured in the cell culture supernatants, thereby allowing us to follow the expression kinetics from the same well of transfected cells after a single transfection. In the case of intracellular luciferase, cell lysates would have been needed at every time point and expression kinetics data would have been influenced by transfection efficiencies of different wells. For these reasons, *Metridia* luciferase was selected as marker gene. Furthermore, other studies have also reported about the advantages of *Metridia* luciferase for high throughput screening for secreted proteins^26^ and it’s sensitivity compared to other reporters^27^. To gain insights into the mechanisms underlying the observed increased protein amount(s) with CYBA UTRs, experiments were performed to determine the mRNA stability of different CYBA UTR containing mRNA constructs.

### Determination of mRNA decay kinetics via quantitative RT-PCR and mRNA productivity

**Figure 4:**
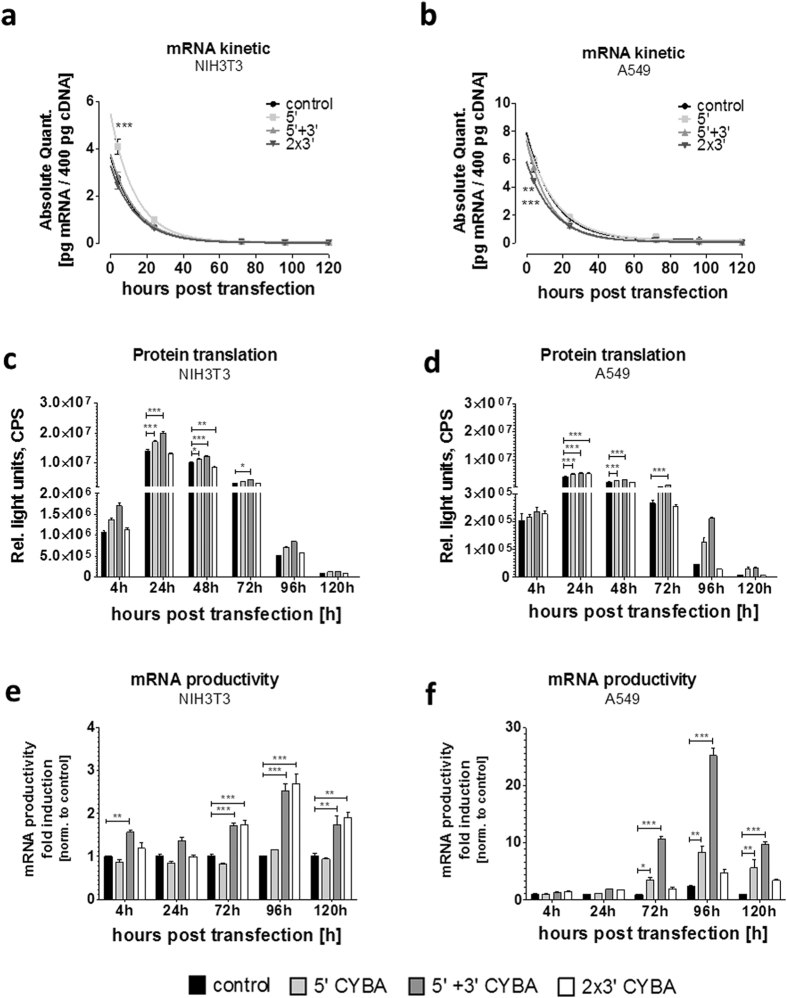
Determination of mRNA quantity, the corresponding protein amounts and the resulting mRNA productivity (*Met*Luc). mRNA decay kinetics in NIH3T3 (**a**) and A549 cells (**b**). mRNA amounts over time were quantified by qRT-PCR. The corresponding *Met*Luc protein translation data at 4, 24, 48, 72, 96 and 120 hours post-transfection in NIH3T3 (**c**) and in A549 cells (**d**). Mean fold-induction of transcript productivity of the different CYBA UTR combinations in NIH3T3 (**e**) and A549 (**f**) cells, respectively. Data represent means ± standard error of the mean (SEM) of three replicates. Statistical significance was assessed by 2-way ANOVA test with p values: *p < 0.5, ** p < 0.01, *** p < 0.001.

Based on UTR screening results (Figs 2 and 3), *Met*Luc-CYBA 5 + 3 recombinant mRNA construct produced the best increase in protein levels compared to the other tested cellular UTRs in both NIH3T3 and A549 cells. Therefore, this *Met*Luc-encoding mRNA construct together with two other constructs of CYBA UTR, were selected for physical mRNA stability analysis and were investigated via quantitative RT-PCR. mRNA decay data were obtained and extrapolated as a half-life by fitting the one-phase decay. The 5′-CYBA UTR is not known to bear any identifiable motifs that may enhance protein translation. For 3′-UTRs, Holtkamp *et al*. have shown that using two copies of the 3′-UTR used in tandem, enhanced protein production^18^. Therefore, the tested construct set included 5′-, 5 + 3 and 2X3 CYBA UTR. Interestingly, we could not observe any stabilizing effect on the mRNA decay kinetics by CYBA UTRs in contrast to the control (without UTRs), with the exception of 5′-CYBA transcript at 4 h post-transfection, in both cell lines (Fig. 4a,b). The mRNA decay kinetics of CYBA UTR bearing constructs was comparable to the decay kinetics of the construct without UTRs. Furthermore, for all combinations (5′-, 5 + 3 and 2X3), no significant increase in the physical T_1/2_ of *Met*Luc-CYBA recombinant transcripts was observed compared to the control mRNA in either cell line (Supplementary Fig. S3a,b and Supplementary Table S2). For the same samples, *Met*Luc activity in NIH3T3 and A549 cells was measured and data is presented as Fig. 4c and d, respectively. No significant differences in *Met*Luc protein levels could be observed for the tested UTRs compared to the control at 4 h post-transfection in either cell line indicating comparable translation initiation rates for the compared constructs. At later time points (>24 h), increased total protein amounts were observed in both cell lines with *Met*Luc mRNA carrying the 5 + 3 CYBA UTRs (Fig. 4c,d). As similar mRNA amounts over time were observed for all mRNA constructs including the control, but higher protein levels for different CYBA UTR containing mRNAs, higher mRNA productivity, defined as the amount of protein (*Met*Luc values) normalized to the amount of mRNA (quantified via qRT-PCR), was observed for mRNA structures furnished with CYBA UTRs (Fig. 4e,f). For each time point and sample, mRNA productivity was calculated by dividing the protein amounts (*Met*Luc) through mRNA amounts. This value was then normalized to the corresponding value observed for control RNA (without UTRs) and presented as “fold change compared to control”. The single mean values of RNA amount, protein amount and calculated mRNA productivity for each transcript at each time point in NIH3T3 and A549 are shown in Supplementary Tables S3 and S4, respectively.

RNA productivity is likely to be a better mark for transcript therapeutics as it allows the comparison of protein, the actual therapeutic product, resulting after delivery of a specific dose of transcript. Taken together, use of CYBA UTRs in recombinant transcripts significantly enhances their productivity without affecting their stability. Depending on the UTR combination, these effects seem to be cell-type specific, with the exception of the 5 + 3 CYBA UTR combination.

**Figure 5:**
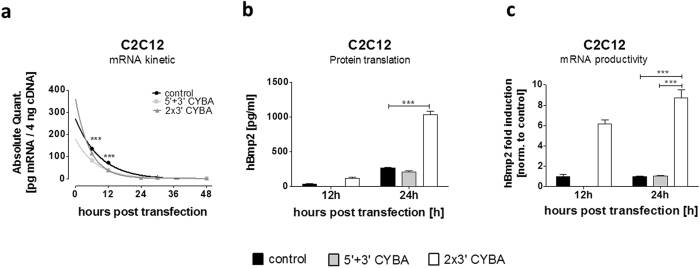
Determination of the hBMP2 mRNA decay kinetics via qRT-PCR, quantification of hBMP2 protein and mRNA productivity. C2C12 cells were transfected with hBMP2-CYBA 2 × 3 and hBMP2-CYBA 5 + 3 transcripts and were compared to the control without UTRs. Absolute mRNA quantification (**a**) as well as the corresponding protein amounts (**b**) were determined by qRT-PCR and ELISA, respectively. Mean fold induction of the mRNA productivity of the different CYBA UTR combinations at 12 and 24 hours post-transfection (**c**). Data represent means ± SEM of three replicates. Statistical significance was assessed by 2-way ANOVA test with p values: ***p < 0.001.

**Figure 6:**
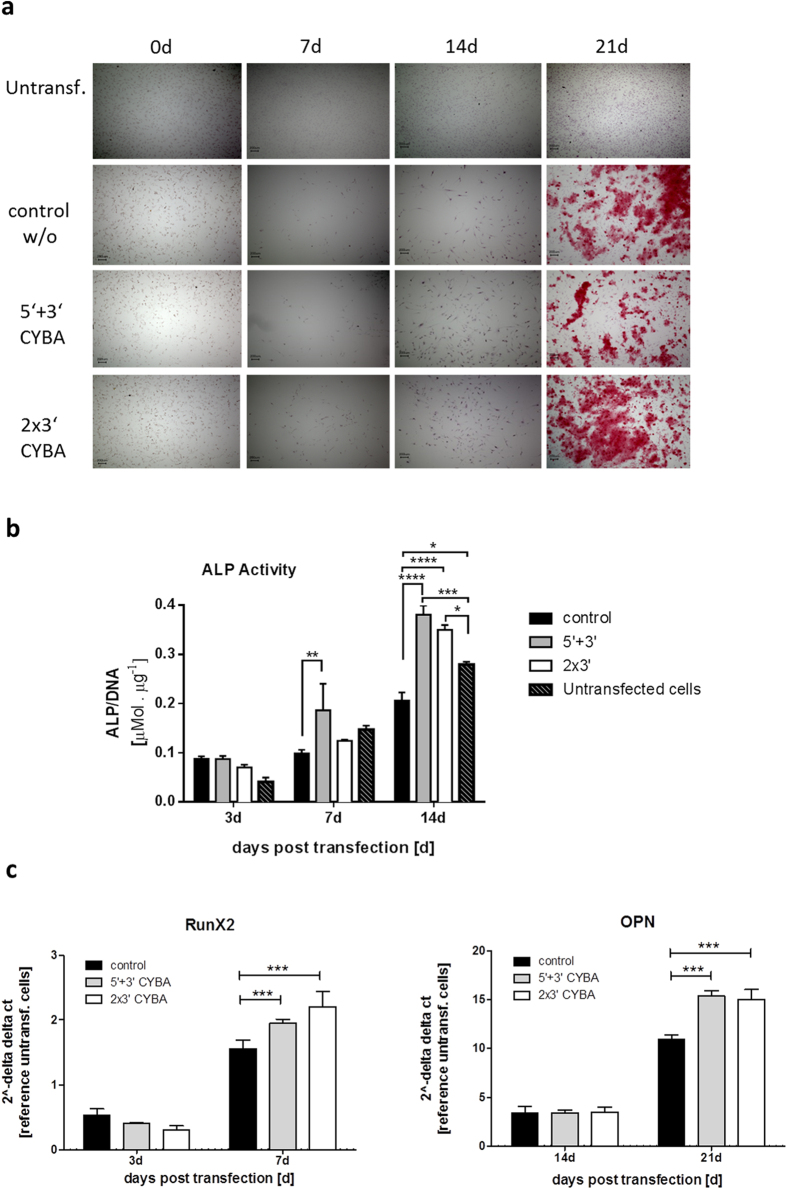
Osteogenic differentiation of hAMSC cells after transfection with hBMP2 transcripts as confirmed by alizarin red staining, ALP activity and qRT-PCR of RunX2 and OPN. (**a**) Mineralization of hAMSCs after recombinant hBMP2-CYBA mRNA transfection was visualized after 0, 7, 14 and 21 days using alizarin red staining. The scale bars represent 200 μm. (**b**) ALP activity was quantified at 3, 7 and 14 days post-transfection (**c**) Total RNA was extracted and qRT-PCR was performed at 3 and 7 days for RunX2 and 14 and 21 days for OPN after transfection. Expression is reported as fold induction compared to untransfected controls. All values were normalized to the housekeeping gene ß-tubulin. Data represent means ± SEM of three replicates. Statistical significance was assessed by 2-way ANOVA test with p values: *p < 0.5, **p < 0.01, ***p < 0.001, ****p < 0.0001.

Experiments with *Met*Luc demonstrated that some of the tested recombinant mRNA constructs furnished with CYBA UTR combinations resulted in higher protein levels compared to the control without UTRs. *Met*Luc is a reporter protein from non-mammalian organisms. In subsequent experiments, effects of CYBA UTR(s) on the transcript T_1/2_ and productivity using a mammalian sequence were investigated. Human BMP2, which plays an important role in bone and cartilage development, was chosen for these experiments. Therefore, all different combinations of CYBA UTRs were cloned upstream/downstream of hBMP2 coding sequence. Agarose gel electrophoresis was used to check the integrity and quality of IVT- mRNA (Supplementary Fig. S4a). The control construct without any UTRs in Supplementary Fig. S4 and subsequently in Figs 5 and 6 refers to the transcript used by Balmayor *et al*.^22^.

After production, the transcripts were transfected into murine C2C12 cells. C2C12 cells are derived from thigh muscle of C3H mice and are frequently used for differentiation into myoblast and osteoblast cell lineages^28^,^29^,^30^,^31^. Hence this cell line was selected for screening purposes of recombinant hBMP2 mRNAs furnished with all combinations of CYBA UTR.

For these cells, a standard protocol comprising of an *in vitro* transfection lipid in conjunction with magnetofection was used to increase transfection efficiency^22^. Magnetofection uses a magnetic field to enhance the transfection of cells with nucleic acids that are complexed with magnetic beads and it has been also shown to be an efficient tool for inducing osteogenic differentiation^22^,^32^,^33^,^34^.

As a result, the inclusion of CYBA UTRs in a hBMP2 transcript increased the production of hBMP2 in C2C12 cells especially at 48 h (Supplementary Fig. S4b). Presence of the CYBA 3′-UTR had a stronger effect than the 5′-UTR. Transcripts carrying two copies of CYBA 3′-UTR flanking hBMP2 resulted in the highest levels of hBMP2 protein compared to all other UTR combinations.

### Determination of hBMP2-CYBA recombinant transcript T_1/2_ and total hBMP2 protein production in C2C12 cells

Results obtained from the magnetofection of C2C12 cells indicated that hBMP2-CYBA 2X3 transcripts resulted in highest, while hBMP2-CYBA 5 + 3 transcripts resulted in the lowest hBMP2 protein levels (Supplementary Fig. S4b). Both of these recombinant transcripts were selected for further stability analysis via qRT-PCR. hBMP2 transcripts bearing CYBA UTRs did not result in any increased T_1/2_ by comparison to control hBMP2 transcript without UTRs (Fig. 5a and Supplementary Fig. S3c and Supplementary Table S2). Rather a significant decrease in the stability of hBMP2- CYBA 2X3 transcripts was detected. Unexpectedly, while a decrease in hBMP2-CYBA 2X3 transcripts was observed, the same transcript revealed the highest protein level (Fig. 5b) and the highest mRNA productivity than either the control or the hBMP2-CYBA 5 + 3 transcripts (Fig. 5c). In contrast, no hBMP2 protein was observed at 12 h for hBMP2-CYBA 5 + 3 transcripts despite the fact that comparable levels of transcripts were observed using qRT-PCR measurement (Fig. 5a). It is conceivable that a delay in translational initiation of the hBMP2-CYBA 5 + 3 construct occurs as compared to the other two hBMP2 constructs. The mean values of RNA amount, hBMP2 concentration and calculated mRNA productivity for each transcript at each time point in C2C12 cells are shown in Supplementary Table S5. Measurements of hBMP2 protein revealed that the presence of two copies of 3′CYBA UTR significantly increased mRNA productivity (6–8 fold) over either the hBMP2 control or the hBMP2-CYBA 5 + 3 transcript at the 24 h time point (Fig. 5c). To conclude, insertion of 2 copies of 3′UTR from CYBA downstream of the coding region enhanced total protein production as compared to the control lacking such UTRs.

### hBMP2 transcripts carrying CYBA UTRs promote osteogenic differentiation of hAMSCs

Next, the osteogenic potential of hBMP2-CYBA transcripts versus the control hBMP2 transcript without UTRs in hAMSC cells was assessed *in vitro* by alizarin staining. The control without CYBA UTRs represents the standard hBMP2 transcript used by Balmayor *et al*.^22^. Cells were transfected at a dose of 20 pg/cell using magnetofection and at 21 days post-transfection of hBMP2 transcripts, calcified nodules could be clearly detected in transfected cells (Fig. 6a). Alkaline phosphatase (ALP) is another marker for *in vitro* osteogenesis. ALP activity at 3, 7 and 14 days post-transfection of hAMSC cells with different hBMP2 transcripts was quantified. As cells continue to divide post transfection, for normalization of ALP values, total DNA (as a measure of cell proliferation) was isolated and ALP values were normalized to DNA isolated from the entire well. As shown in Fig. 6b, significant increase in ALP activity for hBMP2-CYBA UTR bearing transcripts in contrast to the control without UTRs at 14 days post-transfection was observed. Similar to ALP acitivity, expression of osteogenesis-related genes including RunX2 and OPN was quantified at different time points by real time PCR (Fig. 6c). Significantly enhanced expression of RunX2 and OPN was observed for cells transfected with CYBA-bearing transcripts as compared to the control. To summarize, it could be clearly shown that incorporation of CYBA UTRs resulted in higher mRNA productivity which in turn translated to significantly higher expression of bone differentiation markers Runx2, OPN and ALP when compared to the control BMP2 RNA without UTRs.

## Discussion

In this study five different cellular UTR sequences (CYBA, DECR1, GMFG, MAPBPIP and MYL6B) based on mRNA stability data were selected and were investigated with respect to translation and mRNA stability. None of these selected UTR sequences are housekeeping genes. The goal of this study was to investigate whether UTRs from long-lived cellular transcripts would be able to confer increased stability and translation efficiency when combined with other gene sequences. To that end, we designed hybrid mRNA molecules consisting of a selected set of 5′- and 3′-UTR combinations and chemically modified nucleotides. The area under the curve (AUC), specifying the total protein amount over a period of time, was quantified to determine the best productive UTR combination(s).

In fact, only a few of the tested UTRs and their combinations resulted in increased total protein amounts as compared to the control without UTRs. Hybrid transcripts containing CYBA UTRs yielded the highest *Met*Luc reporter protein amounts among all the UTRs that were screened in NIH3T3 and A549 cells. In both cell types, hybrid mRNA constructs comprising 5 + 3 CYBA UTRs resulted in the highest relative AUC. Ironically, the insertion of CYBA UTRs enhanced total protein amounts without positively affecting the physical stability of the transfected transcript. The mechanisms underlying the effectiveness of CYBA UTRs as enhancers of total protein production as compared to the other cellular UTRs are not known. The 5′-CYBA UTR has no known regulatory motifs whereas the 3′-CYBA UTR contains a polyadenylation signal (PAS) as well as the insulin 3′-UTR stability element (INS_SCE). It is conceivable that an enhanced recruitment of translational factors occurs for these combinations.

Hybrid transcript T_1/2_ was investigated for the most productive CYBA UTR combinations (5 + 3) and was compared to two additional CYBA UTR variants: 5′ -UTR and 2X3 -UTR. Overall, no increase in transcript T_1/2_ was detected for the tested constructs as compared to the control. Our measurements of physical mRNA stability via qRT-PCR include the entire population of translated and untranslated mRNA whereas any measurement of improvements in total protein amounts can be viewed as increases in therapeutic potential or “functional half-life” (a measure of productivity). Micro-pattern based single-cell arrays allow the estimation of the functional half-life in a high-throughput manner^35^,^36^. Using these arrays, similar productivity enhancing effect of CYBA UTRs in A549 and Huh7 cells has been previously reported^35^.

Our previous study using destabilized EGFP as a marker gene and single cell arrays was the first report showing that incorporation of CYBA UTRs resulted in higher protein amounts without affecting mRNA stability^35^. There, we could also show that CYBA UTRs had no effect on translation initiation and protein half-life. Due to the technical limitation of single cell arrays, it is not possible to determine expression kinetics for longer than 25 hours. The present study with *Metridia* luciferase allowed us to determine expression kinetics for up to 120 hours (5 days) post-transfection and hBMP2 served as a physiological gene for proof-of-concept studies. These results together with our previous work^35^ present “functional half-life” as a reliable parameter for determining the effectivity of transcript therapies. Different sequences (destabilized EGFP^35^, *Metridia* luciferase and hBMP2) in various cell types, allow us to conclude that incorporation of CYBA UTRs increases the functional half-life of mRNA without affecting it’s physical stability independent of the mRNA sequence.

Although physical mRNA stability was not increased for the *Met*Luc-CYBA construct, some recombinant mRNA bearing CYBA UTRs constructs resulted in enhanced total protein amounts at later time points as compared to controls. One explanation is an increase in mRNA transcript productivity, which we define as the normalization of protein/mRNA ratio and use it to describe the translational capacity of a hybrid transcript. As a consequence, the highest mRNA productivity of *Met*Luc constructs was achieved when 5 + 3 CYBA UTR was tested in NIH3T3 and A549 cells. Two other constructs that delivered high levels of protein per mRNA molecule were 2X3 CYBA UTR and the 5′-CYBA UTR. However, the amount of total protein produced can also depend on mRNA bound to polysomes. Moreover, translation can vary from cell to cell and is not solely depending on the mRNA half-life. Therefore, additional studies are needed to investigate whether ribosome binding or the transition from initiation to elongation phase of translation is altered by the presence of CYBA UTR sequences.

All combinations of CYBA UTRs were cloned upstream and/or downstream of the human hBMP2 mRNA sequence and screened in C2C12 cells (Supplementary Fig. S4b). In contrast to the results with *Met*Luc, hybrid transcript combinations of 5 + 3 CYBA UTR flanking hBMP2 did not result in the highest levels of hBMP2 protein in C2C12 cells. Instead, the highest hBMP2 protein was detected for the recombinant mRNA construct furnished with 2X3 CYBA UTR (Fig. 5). The beneficial effect of two copies of the same UTR has been reported previously by Holtkamp *et al*.^18^.

All these observations together with *Met*Luc-CYBA 5 + 3 transcript being the best in NIH3T3 and A549 whereas hBMP2-CYBA 2X3 transcript being sufficient in C2C12 cells, seem to indicate cell-specific effects contributing to the functional output of hybrid CYBA-bearing constructs. Thus, changes in total protein production may vary in different cell types and when different therapeutic genes are tested^37^,^38^,^39^,^40^. Baudouin-Legros and colleagues have also observed similar effects^39^. They could show that the stability of CFTR (cystic fibrosis transmembrane conductance regulator) transcripts is cell-specific. Endogenous CFTR transcripts are regulated by cytokines such TNFα which bind to the 3′ -UTR. In the presence of TNFα, CFTR mRNA levels in human HT-29 colon cells were decreased but levels were not changed in pulmonary Calu-3 cells^39^. Furthermore, Holtkamp *et al*., have shown that 2 copies of 3′ UTR from β-globin result in increased protein levels and mRNA stability only in immature dendritic cells, whereas in mature dendritic cells, no effect could be observed^18^. Cell specificity of UTRs when combined with mRNA has been also observed for other cells and tissues and has been discussed in the recent reviews^41^,^42^.

Finally, the osteogenic potential of the cmRNA constructs to induce bone differentiation was assessed in hAMSCs. As shown in Fig. 5, incorporation of CYBA UTRs significantly increased the productivity of hBMP2 transcripts. This was also reflected in a higher physiological response to expressed hBMP2 where higher levels of Runx2, OPN and ALP were produced after cells were transfected either with 5 + 3 or 2X3 CYBA UTR combinations (Fig. 6). The construct without UTR, even though resulted in hBMP2 expression (Supplementary Fig. S4b), a positive Alazarin red staining (Fig. 6a) and enhanced RunX2 and OPN expression, did not result in any higher ALP activity. Instead unexpectedly lower ALP values were observed when compared to untransfected cells. The underlying reason for this unexpected result is not known. Expression of ALP in untransfected cells is not unexpected as all cells (both transfected and untransfected) were cultivated in a medium containing ascorbic acid and β-glycerophosphate (Fig. 6b). Both ascorbic acid and β-glycerophosphate are known supplements to supoport osteogenic differentiation in MSCs^43^. Moreover, cells were cultured at low serum conditions to promote differentiation. To summarize, it could be clearly shown that incorporation of CYBA UTRs resulted in higher mRNA productivity which in turn translated to significantly higher expression of bone differentiation markers Runx2, OPN and ALP when compared to the control BMP2 RNA without UTRs.

Previous study from our group has established the proof-of-concept that chemically-modified hBMP2 mRNA promotes osteogenesis of stem cells and enhances bone healing in rats^22^. Nonetheless, those studies were performed using the control RNA (without UTRs) which we now know is much less effective compared to it’s CYBA UTR containing counterparts. These new recombinant hBMP2 mRNAs are most likely to reduce the total dose needed to attain desired bone regeneration thereby reducing the possible unknown side effects and costs of therapy.

Furthermore, the present study was aimed at enhancing the stability of non-immunogenic chemically-modified RNAs by incorporating UTRs from highly stable cellular mRNAs. In contrast to the expectations, none of the 5 tested cellular UTRs in any combination resulted in any increase in the “physical half-life” of mRNA (as determined by real time PCR). Though the underlying mechanism has not been investigated in the current study, following factors either individually or in combination might attribute to the current results.

Firstly, mRNA transcription normally happens in the cell nucleus and mRNA is exported from the nucleus as a ribonucleoprotein complex. Under those conditions, the selected UTR sequences are most likely to have a different conformation and/or bound by RNA binding proteins when compared to the “naked mRNA” produced via *in vitro* transcription and subsequently transfected into the cells. This difference may account for the lack of “physical stability” effects observed in the current study. Another factor which might be playing a role is the use of modified nucleotides in the *in vitro* transcription reaction to produce stable non-immunogenic mRNA. A recent study by Thess *et al*.^17^ has shown that replacement of uridine via pseudouridine resulted in a complete loss of function for EMCV IRES. If and how do the specific modified nucleotides used in the current study affect the functions of tested UTRs remains unknown but will be the subject of further studies. Last but not the least, we are comparing two different quantification methods for mRNA stability. In our study we used qRT-PCR to measure the physical half-life of our CYBA UTR containing RNAs whereas the half-life of the natural CYBA mRNA was assessed by using Affymetrix expression arrays^24^. A direct comparison of both methods concerning the physical half-life of the molecules is not completely given, since the use of primers in our qRT-PCR assay differed from that chosen for microarray probe selection as well as the probe location. Differences in mRNA abundance values determined using different methods (qRT-PCR and microarrays) have been described previously^44^,^45^,^46^.

Another assumption is the influence of secondary structures on the mRNA translation. It is known that mRNA folding starts from the 5′-end and secondary structure in the 5′-UTR of the mRNA either enhances translation or can have inhibitory effects on translation^47^. Mfold-based analysis allows one to determine the thermal stability (Δ)G of predicted secondary structures. No conserved secondary structures could be observed for CYBA UTRs. The ΔG values instead were more dependent on the transgene (*Met*Luc, destabilized EGFP, hBMP2).

Besides secondary structures, GC content has also been reported to affect mRNA stability and translation. Babendure *et al*. reported that high GC content, negatively influences the translational efficiency in cells, independent of thermal stability^48^. In line with this school of thought, we also investigated if any correlation could be observed between GC content of all tested UTRs and their respective total protein amount. CYBA UTRs with highest GC content (Supplementary Table S7) compared to the others are expected to result in lowest protein amounts, which was not the case. Therefore, the mechanism supporting enhanced productivity of CYBA UTR furnished mRNAs remains unknown.

To conclude, CYBA UTRs and their combinations have been found to be a potential candidate for enhancing the productivity of mRNA transcripts thereby establishing the therapeutic potential of hybrid transcript design. The mechanisms underlying the observed increased protein productivity of transcripts furnished with CYBA UTRs still need to be investigated. Such mechanistic studies are likely to identify the currently unknown regulatory motifs in CYBA UTR sequences.

## Methods

### Plasmid preparation

The UTR sequences were cloned into the backbone pVAX1-A120 which has been described previously by Kormann *et al*.^19^. In addition, reporter gene coding for *Medtridia* luciferase (*Met*Luc) was cloned into this backbone between *BamH*I-*EcoR*I sites by Geneart. The resulting plasmid “pVAXA120-*Met*Luc” was used for all further UTR cloning procedures.

The sequences of 5′- and 3′ -UTRs of each of the listed human gene (Supplementary Table S6) were cloned in five different combinations, namely 5′-UTR and 3′-UTR alone as well as 5′ + 3′ UTR, 5′-UTR with 2 copies of 3′-UTR in direct repeats (5 + 2x3 UTR) and two copies of 3′-UTR without 5′-UTR (2x3′ UTR). All of these constructs were compared to a control without UTRs. For the 5′ –UTR containing plasmids, the 5′-UTRs were cloned into pVAXA120-*Met*Luc between *HindIII*-*BamH*I sites upstream of the gene of interest (GOI). For 3′-UTRs, cloning into *EcoR*I-*Pst*I sites was performed.

### Generation of modified mRNA

To generate *in vitro* transcribed mRNA (IVT-mRNA), plasmids were linearized downstream of the poly(A) tail by *Xba*I digestion and purified by chloroform extraction and ethanol precipitation. Purified linear plasmids were used as template for *in vitro* transcription using RiboMax Large Scale RNA production System-T7 (Promega, Germany). Anti-Reverse Cap Analog (ARCA) was added to the reaction mix to generate 5′ capped mRNA. Additionally for the production of cmRNAs, modified nucleotides, methyl-CTP and thio-UTP (Jena Bioscience, Germany), were added into reaction as described by Ferizi *et al*.^35^.

### Cell Culture

A human alveolar adenocarcinoma cell line (A549, ATCC CCL-185) was grown in Minimum Essential Media (MEM) supplemented with 10% fetal bovine serum (FBS) and 1% Penicillin/Streptomycin (P/S). Murine fibroblast cell line (NIH3T3, ATCC CRL-1658) and murine myoblast cell line (C2C12, Sigma Aldrich) as well as human AMSCs were cultured in Dulbecco’s Modified Eagle Medium (DMEM) low glucose, supplemented with 10% FBS and 1% P/S. All cell lines were grown in a humidified atmosphere at 37 °C and 5% CO_2_ level.

### *In vitro* transfection for screening studies

To perform screening experiments, NIH3T3 and A549 cells were transfected with different doses of mRNA/well to evaluate dose dependent effects. The experimental set-up was as follows: 5 × 10^3^ NIH3T3 cells or 7 × 10^3^ A549 cells in 150 μl complete medium were seeded per well in 96-well plates and transfected 24 hours post-seeding, respectively. Cells were transfected at a starting dose of 500 ng/well using the commercial transfection reagent Dreamfect Gold (DFG). Complexes were prepared at a ratio of 4 μl DFG per 1 μg mRNA. The mRNA (3.6 μg) was diluted separately in DMEM without supplements in a reaction tube with a total volume of 340 μl for each mRNA. 14.4 μl DFG was mixed separately with 5.6 μl water for each mRNA complex. Complex formation took place when the mRNA dilution was added to the DFG solution followed by 20 min incubation time at RT. After incubation time, a serial dilution (1:2) was performed. Finally, 50 μl of the complex solution were added onto the cells and incubated for 4 hours. For every mRNA construct, triplicates were prepared. After 4 hours, the complete supernatant was removed from the cell culture plate for further analysis and fresh 200 μl medium was added to each well. This procedure was performed for all following measuring time points, thereby negating any accumulation of *Met*Luc over time. Bioluminescence was measured after 4, 24, 48, 72, 96 and 120 hours using a multilabel plate reader. For this, 50 μl of cell culture supernatant was mixed with 20 μl coelenterazin (50 mM) and the generated light was measured.

### Transfection of C2C12 via DFG and magnetofection

C2C12 cells were transfected with mRNA coding for hBMP2 with and without CYBA UTRs using magnetofection. Before transfection, 5 × 10^4^ cells/well were seeded in 1 ml DMEM complete medium in a 24 well cell culture plate. Cells were transfected at a dose of 20 pg/cell at a ratio of 4 μl DFG per 1 μg recombinant mRNA. Magnetofection was performed by using SoMag5 magnetic nanoparticles. Further information related to the composition and characterization of these magnetic nanoparticles can be found elsewhere^49^. These particles were used at a ratio of 1 μg mRNA per 0.5 μg Fe. Complex formation was prepared by mixing DFG, magnetic nanoparticles and mRNA to a final concentration of 10 μg/ml followed by 20 minutes incubation time at RT. After incubation the complex was further diluted to its working solution with DMEM without supplements. 250 μl of the complex solution was transferred onto the cell containing 250 μl complete medium to a final volume of 500 μl. A magnetic field by using a special made 24 well magnetic plate, was applied under the cell culture plate for 30 minutes at 37 °C. Complete medium change was performed after 24 h post-transfection, thereby negating any accumulation of hBMP2 over time. Human BMP2 translation was measured after 24 hours and 48 hours post-transfection using ELISA.

### RNA isolation and reverse transcription

In order to determine the actual mRNA amount at different time point(s) post-transfection, the cultured cells were lysed and RNA was isolated according to the manufacturer’s protocol using Nucleo Spin RNA kit (Macherey Nagel). The isolated RNA was eluted in 40 μl RNAse free water and was examined for RNA concentration and quality by spectrophotometric measurements and gel analysis, respectively. cDNA was synthesized from isolated RNA (1 μg of transfected NIH3T3 and A549 and 0.5 μg of transfected C2C12 cells, respectively) using First Strand cDNA Synthesis Kit (Thermo Scientific) following the manufacturer’s instruction. The synthesized cDNA was stored at −20 °C.

### Quantitative Real Time Polymerase Chain Reaction (qRT-PCR)

A qRT-PCR based analysis was used to determine the *Met*Luc mRNA amount at time intervals of 4, 24, 48, 72, 96 and 120 hours in A549 and NIH3T3 cells. Additionally, the mRNA expression kinetic itself was used to calculate the mRNA half-life of each UTR. For hBMP2, mRNA was quantified after 6, 12, 24, 30 and 48 hours post-transfection. The reaction was set up as follows: equivalent amounts of 8 μl cDNA (diluted 1:1000 of transfected NIH3T3 and A549 cell and 1:50 of transfected C2C12 cells) were mixed together with 11 μl of Master Mix filled up with water to a total volume of 20 μl. The Master Mix included for one single reaction the following components: 10 μl of Sso Advanced^TM^ Universal SYBR^®^Green Supermix, 0.5 μl of FRW primer and REV primer each (stock solution 20 μM for *Met*Luc primers and 5 μM for hBMP2 primers, respectively). The primers for *Met*Luc and hBMP2 are listed in Supplementary Table S8. Primer amplification efficiency was around 2 and was found to be appropriate for qRT-PCR measurement using LightCycler^®^96 (Roche). For absolute quantification a standard curve was prepared from cDNA from IVT-mRNA.

### hBMP2 ELISA

Cell culture supernatant of transfected cells was used for hBMP2 quantification by ELISA following instructions of the DuoSet^®^ELISA Development system (R&D Systems).

### Transfection of cmRNA hBMP2 into human AMSCs

*In vitro* transfection of mesenchymal stem cells from human adipose tissue was performed following the magnetofection protocol shown previously with slight modifications. Cells were transfected with 20 pg/cell for each mRNA construct in triplicates. At time point of transfection hAMSCs had DMEM without supplements. At 6 hours post-transfection, culture medium was changed to 1 ml/well osteogenic medium (cell culture medium containing 5% FBS, 10 mM β-glycerophosphate, 200 μM ascorbic acid) and was performed every third day until measurements were conducted. Thereby, the media was completely changed with freshly new added 1 ml osteogenic media.

### Alizarin red staining

Mineralization of hAMSCs was evaluated by alizarin red staining according to Balmayor *et al*.^22^. Alizarin red staining was performed 0, 7, 14 and 21 days post-transfection to evaluate calcium deposits in the cells transfected with the hBMP2 cmRNA complex.

### Alkaline phosphatase activity

ALP activity assay is based on the use of p-nitrophenyl phosphate (pNPP) as a phosphatase substrate. pNPP is dephosphorylated in the presence of ALP resulting in a yellow p-nitrophenol (pNP) compound. Alkaline phosphatase activity was evaluated at days 3, 7 and 14 days post-transfection. Briefly, the cells were washed twice with DPBS and incubated with ALP substrate solution (0.2% 4-nitrophenyl phosphate, 50 mM glycine, 1 mM MgCl2, 100 mM TRIS, pH 10.5) for 30 min at 37 °C. The formation of 4-nitrophenol as indication of ALP was photometrically quantified at 405 nm. ALP activity was calculated according to a 4-nitrophenol standard curve. The results were expressed normalized to total DNA amount. For this DNA amount was determined by means of a PicoGreen assay following manufacturer’s instructions (Invitrogen, Molecular Probes). Triplicates were evaluated in all cases.

### Quantitative real-time PCR measurement of osteogenic markers

Expression of osteo-related genes was evaluated at days 3, 7 days for transcription factor RunX2 and 14 and 21 days for OPN post-transfection. Cells were lysed with TRIzol (Life technology, CA, USA). Total RNA was isolated based on phenol/chloroform method. RNA concentration and purity were determined spectrophotometrically. First-strand cDNA was generated using First Strand cDNA Synthesis Kit according to the manufacturer’s instructions. The expression of RunX2, OPN was determined by means of RT-PCR. Human amplification primers are listed in Supplementary Table S8. SsoFast Eva Green Supermix (Bio-Rad Laboratories Inc., CA, USA) was used and real time PCR was carried out on a Bio-Rad CFX96 thermal cycler (Bio-Rad Laboratories Inc., CA, USA). Human ß-tubulin was selected as a housekeeping gene. Data were expressed as fold induction relative to untransfected cells.

### Calculation of area under the curve (AUC)

Luciferase chemiluminescence was measured at several time points post-transfection and AUC was calculated using GraphPad Prism. To achieve the total peak area, the raw data (luciferase values: data not shown) were inserted into a XY table generating a curve (X = time points; Y = luciferase values) for each dose. In Prism, a curve is simply a series of connected XY points. Prism computes the area under the curve using the trapezoid rule. The area, therefore, is ΔX * (Y1 + Y2)/2. Prism uses this formula repeatedly for each adjacent pair of points defining the curve.

## Additional Information

**How to cite this article**: Ferizi, M. *et al*. Human cellular CYBA UTR sequences increase mRNA translation without affecting the half-life of recombinant RNA transcripts. *Sci. Rep.* **6**, 39149; doi: 10.1038/srep39149 (2016).

**Publisher's note:** Springer Nature remains neutral with regard to jurisdictional claims in published maps and institutional affiliations.

## Supplementary Material

Supplementary Information
