# Supplementary material for: Human cellular CYBA UTR sequences increase mRNA translation without affecting the half-life of recombinant RNA transcripts
Source: Sci Rep. 2016 Dec 15;6:39149. doi: 10.1038/srep39149 (PMC5156912; doi:10.1038/srep39149)
Supplement: Supplementary Information [file srep39149-s1.pdf]

Supplementary Information

Human cellular CYBA UTR sequences increase mRNA translation without affecting the half-life of recombinant RNA transcripts

Mehrije Ferizi[1,2], Manish K. Aneja[2], Elizabeth R. Balmayor[3,*], Zohreh S. Badieyan[1], Olga Mykhaylyk[1,2], Carsten Rudolph[2], Christian Plank[1,2,*]

[1]Institute of Molecular Immunology- Experimental Oncology, Klinikum rechts der Isar, Technische Universität München, Munich, 81675, Germany
[2]Ethris GmbH, Planegg, 82152, Germany
[3]Experimental Trauma Surgery, Klinikum rechts der Isar, Technische Universität München, Munich, 81675, Germany
*corresponding authors: Elizabeth.rosado-balmayor@tum.de; christian.plank@tum.de

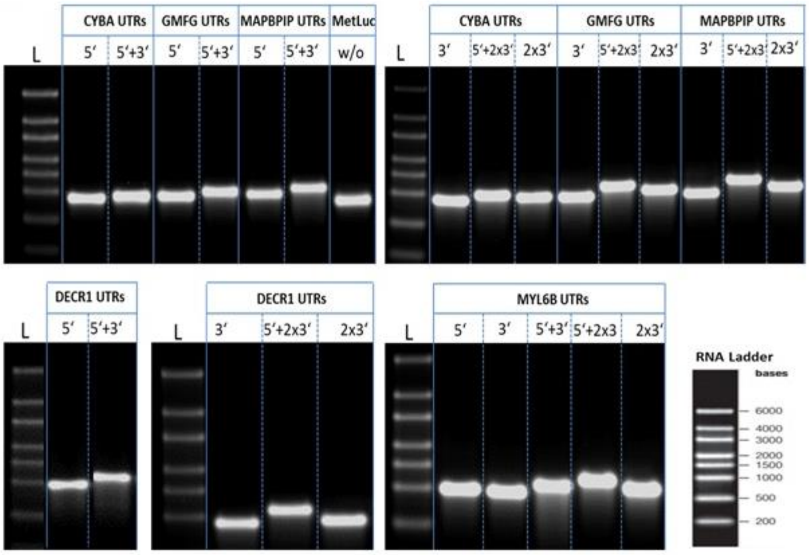

Figure S1: Agarose gel electrophoresis of the different recombinant mRNA products. 1 µg recombinant mRNA of each construct was loaded on 1% agarose gel. RiboRuler High Range RNA ladder (L) was used to determine the correct length of the transcripts (right corner). As shown here, all transcripts showed only one single band as expected.

Size of transcripts coding for *Met*Luc

UTRs	5' UTR	3' UTR	5+3' UTR	5+2x3' UTR	2x3' UTR
CYBA	789 b	800 b	858 b	932 b	874 b
DECR1	975 b	935 b	1082 b	1210 b	1051 b
GMFG	932 b	854 b	1055 b	1114 b	982 b
MAPBPIP	986 b	875 b	1130 b	1210 b	1024 b
MYL6B	977 b	948 b	1097 b	1222 b	1073 b

Table S1: Size of transcripts coding for *Met*Luc. The size, length in bases (b), of each recombinant mRNA construct furnished with cellular UTRs is listed above. Additionally, all produced transcripts showed a single band on 1% agarose gel and were free of protein and organic contamination verified by agarose gel electrophoresis and spectrophotometric measurement, respectively. This recombinant mRNA starting material was used for the entire screening studies *in vitro*.

Screening of different transfection reagents for mRNA delivery

In an initial experiment, different transfection reagents (TfR) were compared for mRNA delivery in NIH3T3 and A549 cell lines. Four different commercially available TfRs namely, Dreamfect Gold (DFG, OzBiosciences), Lipofectamine^{TM}2000 (Invitrogen), MetafectenePro (Biontex) and Dogtor (OzBiosciences) were investigated with respect to the resulting protein translation and cell viability post-transfection. Both transfection efficiency and cell viability were evaluated to compare the different TfR. Cell viability was quantified by MTT assay. In both tested cell lines, MetafectenePro resulted in significant higher protein translation over a broad range of transfected mRNA doses (Figure S2 a, b). Nonetheless mRNA delivery with MetafectenePro resulted in extensive cell death with cell viability falling to below 50% at higher doses in both cell lines. DFG instead showed an overall survival rate of more than 75% in NIH3T3 (up to 500 ng/well) and A549 cells (up to 250 ng/well) compared to MetafectenePro (Figure S2 c, d).

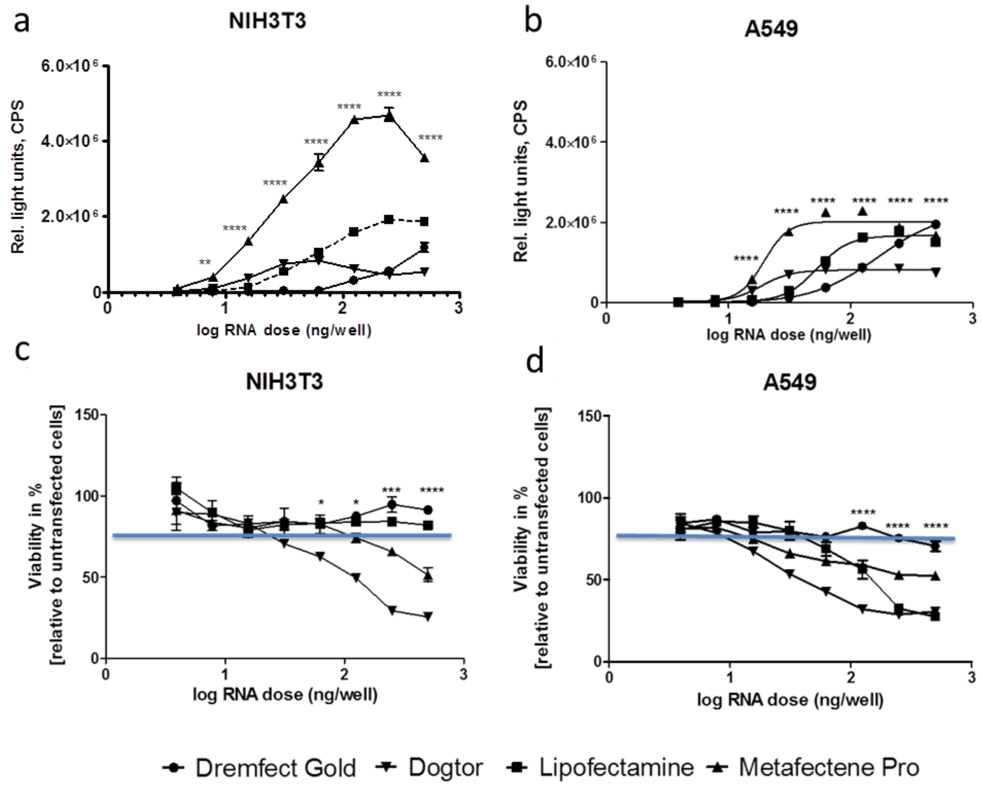

Figure S2: Testing of various TfRs for mRNA delivery with respect to dose-dependent protein translation and cell viability. Recombinant messenger RNA coding for *Met*Luc w/o UTRs was transfected with four different TfRs into NIH3T3 and A549 cells. TfRs screened were DFG, Lipofectamine™2000, MetafectenePro and Dogtor. After 24 hours, *Metridia* luciferase activity (counts per second- CPS) in NIH3T3 (a) and A549 (b) as well as the cell viability of NIH3T3 (c) and A549 (d) were measured. Data represent means (n=3) ± standard error of mean (SEM). Statistical significance was assessed by 2-way ANOVA test with p values: * $p<0.5$, ** $p<0.01$, *** $p<0.001$, ****$p<0.0001$.

Determination of mRNA half-life of *Met*Luc RNA and hBMP2 RNA

To determine the mRNA half-life of *Met*Luc in both cell lines including NIH3T3 and A549, mRNA decay kinetic data were obtained by qRT-PCR (Fig. 4a, b) and were fitted in one-phase decay equation. The same was performed for hBMP2 transcript in C2C12 cells. As a result we could not observe any significant changes in mRNA $T_{1/2}$ of CYBA UTR bearing transcripts in contrast to the control without UTRs (Fig. S3 a, b) with the exception of hBMP2-CYBA 2X3 transcript showing a decrease in mRNA $T_{1/2}$ in C2C12 cells (Fig. S3 c). Mean values of physical mRNA half-life are listed in Table S2.

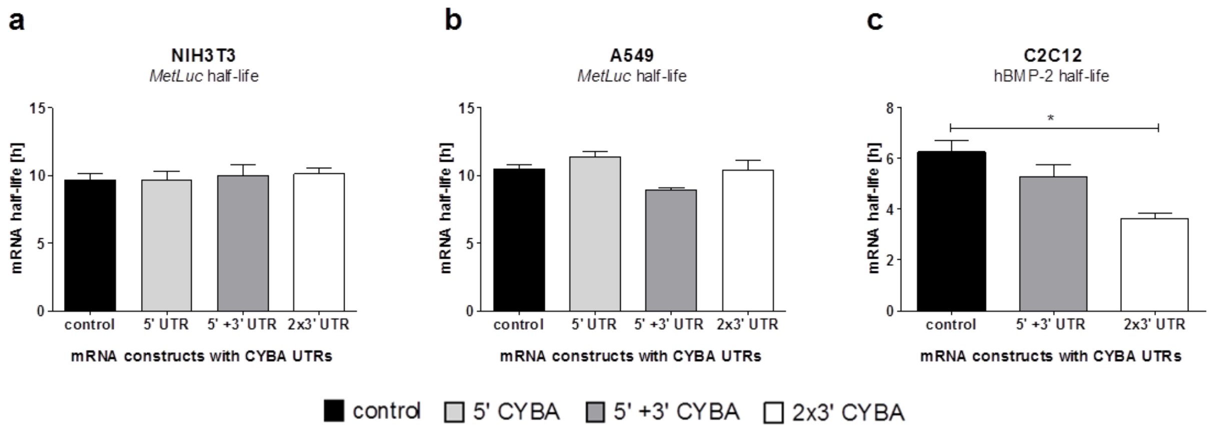

Figure S3: Determination of transcript $T_{1/2}$. mRNA quantification data were used to obtain the mRNA half-life by fitting the one-phase decay. mRNA $T_{1/2}$ of *Met*Luc RNA in NIH3T3 and A549 (b) as well as hBMP2 mRNA half-life in C2C12 cells (c) comparing CYBA UTR bearing constructs to the control without UTRs. Statistical significance was assessed by 1-way ANOVA test with p values <0.05.

mRNA $T_{1/2}$ of various CYBA-UTR containing transcripts in different cell lines

NIH3T3

UTRs	Physical $T_{1/2}$ [h]
**control**	9.64 ± 0.84
**5' CYBA**	9.63 ± 1.16
**5'+3' UTR**	9.98 ± 1.35
**2x3' UTR**	10.10 ± 0.82

A549

UTRs	Physical $T_{1/2}$ [h]
**control**	10.48 ± 0.56
**5' CYBA**	11.39 ± 0.61
**5'+3' UTR**	8.95 ± 0.24
**2x3' UTR**	10.42 ± 1.25

C2C12

UTRs	Physical $T_{1/2}$ [h]
**control**	6.26 ± 0.75
**5'+3' UTR**	5.31 ± 0.76
**2x3' UTR**	3.62 ± 0.43

Table S2: Physical $T_{1/2}$ of various transcripts. Physical mRNA half-life time of different CYBA-UTR furnished transcripts in various cell lines. Mean (±SEM) of three replicates is presented.

Calculation of mRNA productivity

For each time point and sample, mRNA productivity was calculated by dividing the protein amounts (luciferase values [relative light units - RLU] or hBMP2 concentration [pg/ml]) through mRNA amounts (RT-PCR-based quantification [pg mRNA/ pg cDNA]). This value was then normalized to the corresponding value observed for control RNA (without UTRs) and presented as "fold change compared to control" in Fig. 4 of the manuscript. Table S3, S4 and S5 shows the values of RLU, mRNA amount as well as the resulting mRNA productivity at each time point for each mRNA transcript in NIH3T3, A549 and C2C12, respectively.

NIH3T3 RNA concentration [pg mRNA/400 pg cDNA] ± SEM						
	**4 h**	**24 h**	**48 h**	**72 h**	**96 h**	**120 h**
**contr.**	2.75 ±0.15	0.68 ±0.11	n.d	0.07 ±0.01	0.03 ±0.0	0.02 ±0.0
**5’ UTR**	4.09 ±0.57	0.98 ±0.04	n.d	0.10 ±0.01	0.04 ±0.0	0.02 ±0.0
**5’+3’ UTR**	2.83 ±0.29	0.71 ±0.04	n.d	0.06 ±0.0	0.02 ±0.0	0.01 ±0.0
**2x3’ UTR**	2.47 ±0.32	0.63 ±0.12	n.d	0.04 ±0.0	0.01 ±0.0	0.01 ±0.0

NIH3T3 Relative light units [counts per second] ± SEM in %						
	**4 h**	**24 h**	**48 h**	**72 h**	**96 h**	**120 h**
**contr.**	1.07×10^6 ±8.02%	13.93×10^6 ±6.99%	10.02×10^6 ±5.89%	3.06×10^6 ±3.79%	5.13×10^5 ±3.16%	9.46×10^4 ±2.12%
**5’ UTR**	1.37×10^6 ±5.37%	16.99×10^6 ±7.10%	11.19×10^6 ±2.36%	3.63×10^6 ±2.98%	7.15×10^5 ±1.87%	1.17×10^5 ±1.11%
**5’+3’ UTR**	1.71×10^6 ±8.31%	19.88×10^6 ±6.97%	12.12×10^6 ±3.94%	4.29×10^6 ±2.23%	8.48×10^5 ±3.04%	1.37×10^5 ±1.88%
**2x3’ UTR**	1.13×10^6 ±5.82%	12.95×10^6 ±3.37%	8.55×10^6 ±1.86%	3.033×10^6 ±0.94%	5.72×10^6 ±1.44%	8.73×10^4 ±2.21%

NIH3T3 mRNA productivity [RLU/ RNA amount] ± SEM in %						
	**4 h**	**24 h**	**48 h**	**72 h**	**96 h**	**120 h**
**contr.**	3.90×10^5 ±3.05%	20.62×10^6 ±9.95%	n.d	44.96×10^6 ±7.48%	14.68×10^6 ±1.89%	5.99×10^6 ±12.49%
**5’ UTR**	3.36×10^5 ±5.07%	17.34×10^6 ±8.76%	n.d	36.98×10^6 ±13.57%	16.98×10^6 ±8.83%	5.70×10^6 ±9.26%
**5’+3’ UTR**	6.04×10^5 ±12.13%	28.10×10^6 ±9.59%	n.d	77.24×10^6 ±14.30%	36.86×10^6 ±8.67%	10.21×10^6 ±5.85%
**2x3’ UTR**	4.56×10^5 ±10.58%	20.62×10^6 ±10.05%	n.d	78.17×10^6 ±4.68%	38.93×10^6 ±1.08%	11.54×10^6 ±2.89%

Table S3: RNA amount, luciferase expression (Relative light units – RLU) and the resulting mRNA productivity in NIH3T3 cells. For each transcript (CYBA-UTR transcripts and control without UTRs) mRNA amount, protein amount and mRNA productivity were determined at different time points (hours – h; n.d – not determined). Mean values (± SEM) of three replicates are presented.

**A549**<br>**RNA concentration [pg mRNA/400 pg cDNA] ± SEM**						
	**4 h**	**24 h**	**48 h**	**72 h**	**96 h**	**120 h**
**control**	6.07 ±0.28	1.76 ±0.17	n.d.	0.43 ±0.05	0.23 ±0.03	0.09 ±0.02
**5' UTR**	5.94 ±0.22	1.86 ±0.08	n.d.	0.44 ±0.03	0.18 ±0.01	0.07 ±0.01
**5'+3' UTR**	5.42 ±0.52	1.24 ±0.16	n.d.	0.27 ±0.14	0.10 ±0.03	0.04 ±0.02
**2x3' UTR**	4.44 ±0.16	1.21 ±0.14	n.d.	0.20 ±0.03	0.07 ±0.02	0.03 ±0.0
**A549**<br>**Relative light units [counts per second] ± SEM in %**						
	**4 h**	**24 h**	**48 h**	**72 h**	**96 h**	**120 h**
**control**	2.05×10^5 ±16.71	3.93×10^6 ±9.24	2.05×10^6 ±10.14	2.68×10^5 ±5.79	4.70×10^4 ±4.06	7.99×10^3 ±4.16
**5' UTR**	2.16×10^5 ±6.78	4.92×10^6 ±8.40	2.60×10^6 ±5.44	5.04×10^5 ±2.07	1.27×10^5 ±7.85	3.16×10^4 ±6.66
**5'+3' UTR**	2.37×10^5 ±10.70	5.18×10^6 ±7.74	2.89×10^6 ±7.73	9.75×10^5 ±3.74	2.13×10^5 ±3.52	3.54×10^4 ±13.72
**2x3' UTR**	2.29×10^5 ±8.25	5.00×10^6 ±9.43	2.01×10^6 ±7.97	2.56×10^5 ±3.62	2.95×10^4 ±2.52	7.43×10^3 ±0.45
**A549**<br>**mRNA productivity [RLU/ RNA amount] ± SEM in %**						
	**4 h**	**24 h**	**48 h**	**72 h**	**96 h**	**120 h**
**control**	3.37×10^4 ±17.11	2.24×10^6 ±0.95	n.d.	6.26×10^5 ±6.29	2.02×10^5 ±8.79	8.44×10^4 ±15.48

**5' UTR**	3.64×10^4 ±10.46	2.65×10^6 ±5.11	n.d.	1.14×10^6 ±5.98	7.03×10^5 ±3.82	4.64×10^5 ±8.17
**5'+3' UTR**	4.38×10^4 ±10.47	4.17×10^6 ±11.46	n.d.	3.65×10^6 ±2.44	2.15×10^6 ±8.50	8.35×10^5 ±6.29
**2x3' UTR**	5.16×10^4 ±12.09	4.12×10^6 ±1.79	n.d.	1.26×10^6 ±11.53	3.96×10^5 ±13.37	2.97×10^5 ±9.92

Table S4: RNA amount, luciferase expression (Relative light units – RLU) and the resulting mRNA productivity in A549 cells. For each transcript (CYBA-UTR transcripts and control without UTRs) mRNA amount, protein amount and mRNA productivity were determined at different time points (hours – h; n.d – not determined). Mean values (± SEM) of three replicates are presented.

**C2C12 RNA concentration [pg mRNA/4 ng cDNA] ± SEM**					
	**6 h**	**12 h**	**24 h**	**30 h**	**48 h**
**control**	135.28 ±6.26	72.82 ±5.11	9.99 ±0.89	6.26 ±0.18	1.11 ±0.05
**5'+3' UTR**	81.78 ±3.35	37.95 ±4.70	7.51 ±0.15	4.02 ±0.15	1.05 ±0.08
**2x3' UTR**	115.32 ±10.17	37.84 ±7.78	4.44 ±0.47	3.94 ±0.37	1.06 ±0.03
**C2C12 hBMP2 concentration [pg/ ml] ± SEM**					
	**6 h**	**12 h**	**24 h**	**30 h**	**48 h**
**control**	n.d.	36.86 ±12.61	269.61 ±17.02	n.d.	n.d.
**5'+3' UTR**	n.d.	0	211.40 ±25.81	n.d.	n.d.
**2x3' UTR**	n.d.	121.21 ±29.58	1,037.53 ±80.91	n.d.	n.d.
**C2C12 mRNA productivity [hBMP2 pg/ml/ RNA amount] ± SEM**					
	**6 h**	**12 h**	**24 h**	**30 h**	**48 h**
**control**	n.d.	0.51 ±0.16	243.01 ±1.34	n.d.	n.d.
**5'+3' UTR**	n.d.	0	200.78 ±2.72	n.d.	n.d.
**2x3' UTR**	n.d.	3.20 ±0.25	980.39 ±17.32	n.d.	n.d.

Table S5: RNA amount, hBMP2 concentration (pg/ ml) and the resulting mRNA productivity in C2C12 cells. For each transcript (CYBA-UTR transcripts and control without UTRs) mRNA amount, protein amount and mRNA productivity was determined at different time points (hours – h; n.d – not determined). Mean values (± SEM) of three replicates are presented.

Screening of hBMP2-CYBA constructs in C2C12 cells

The integrity of the modified mRNA constructs coding for hBMP2 was checked on 1% agarose gel (Fig. S4a). mRNA magnetofection studies were conducted by using SoMag 5 nanoparticles at a dose of 20 pg mRNA/cell. At 24 and 48 hours post-transfection, hBMP2 was quantified using ELISA. The hBMP2-CYBA 2X3 transcript showed the highest and significant increase in hBMP2 protein amounts at both time points compared to the control (Fig. S4b). Messenger RNA constructs with 3' and 5'+2x3' CYBA UTR also resulted in significantly higher levels of hBMP2 at 48h post-transfection compared to the control. Recombinant mRNA constructs with 5'-UTR alone or hBMP2-CYBA 5+3 resulted in the lowest protein amount compared to the other tested mRNA constructs with CYBA UTR combinations.

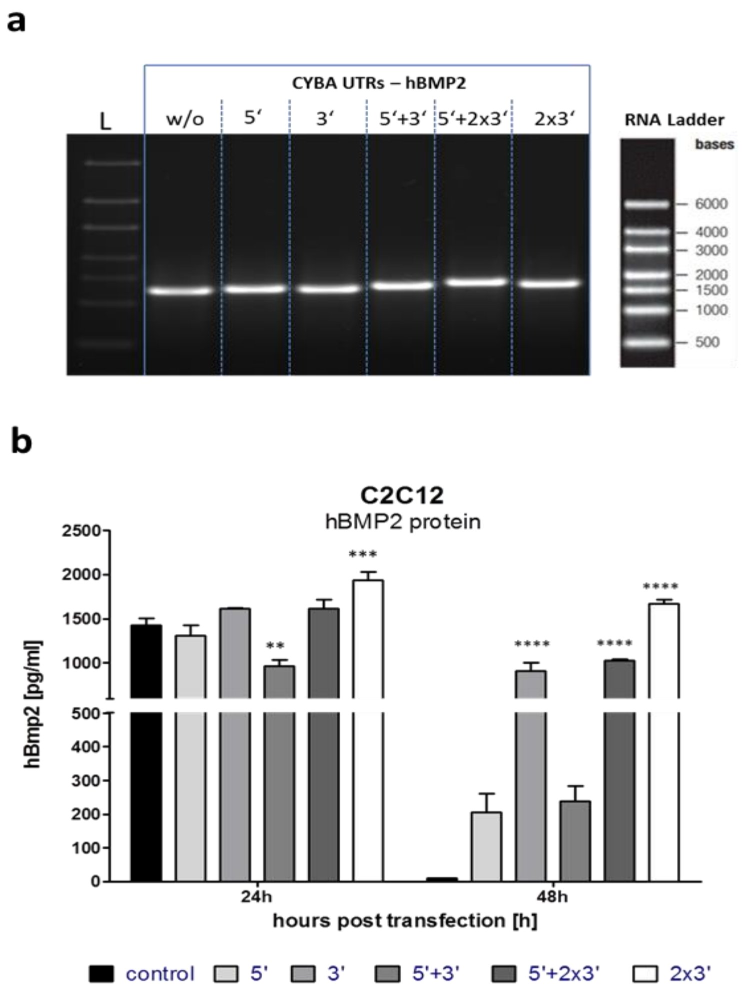

Figure S4: Comparing hBMP2 translation after transfecting different mRNA constructs furnished with CYBA UTRs. The different mRNA constructs were checked on 1% agarose gel. As shown here, all mRNAs indicated only one single band (a). The sizes of the mRNA constructs (bases): 1381 b (hBMP2 w/o UTRs), 1417 b (5’ CYBA UTR), 1476 b (3’ CYBA UTR), 1486 b (5’+3’ CYBA UTR), 1560 b (5’+2x3’ CYBA UTR) and 1490 b (2x3’ CYBA UTR). (b) mRNA coding for hBMP2 with CYBA UTR combinations, including 5’, 3’, 5’+3’, 5’+2x3’ and 2x3’ UTR, were transfected into C2C12 cells. Human BMP2 expression was quantified at 24 hours and 48 hours post-transfection. Values present mean ± standard error of three replicates. Statistical significance was assessed by 2-way ANOVA test with p values: ** $p<0.01$, *** $p<0.001$, ****$p<0.0001$.

Human cellular untranslated regions (UTRs) and their features

UTRs	Human UTR Sequences [from 5' to 3']		Features	
	**5' UTR**	**3' UTR**	**5' UTR**	**3' UTR**
**CYBA**	cgcgcctagcagtgtcccagccgggttcgtgtcgcc (36bp)	cctcgccccggacctgccctcccgccaggtgcacccacctgcaataa**atgcagcgaagccggga** (64bp)	-	INS_SCE; PAS
**DECR1**	acGccgcctgggtcccagtccccgtcccatcccccggcggcctaggcagcgtttccagccccgagaactttgttcttttttgtcccgccccctgcgcccaAccgcctgcgccgccttccggcccgagttctggagactcaac (141bp)	Gaccactttggccttcatcttggttacagaaaagggaatagaaatgaaacaaattatctctcatcttttgactatttcaagtcta**ataaattcttaattaac** (102bp)	SNP (rs67780505; rs72368577)	PAS; SNP (rs7162)
**GMFG**	gttgg*atgaaaccttcctcctactgcacagcccgcccccctacagccccggtccccacgcctagaagacagcggaactaa*gaaaagaagaggcctgtggacagaacaatc (110bp)	tctctgggctggggactgaattcctgatgtctgagtcctcaaggtgactggggacttggaacccctaggacctgaacaaccaagactta**aataaattttaaaatgcaaaaactcgga** (118bp)	uORF;	PAS
**MAPBPIP (ROBLD3)**	ggtggggcggggttgagtcggaaccacaatagccaggcgaagaaactacaactcccagggcgtcccggagcaggccaacgggactacgggaagcagcgggcagcggcccgcgggaggcacctcggagatctgggtgcaaaagcccagggttaggaaccgtaggc (164bp)	cggcattggtggaagctggggtcagaaaagagaaatgaccatttggaggggcggggcctcctagaagaaccttcttagacaatggggggaggatgggactttgttttttccaag**aataaacttcaactcctgtcatgtg** (139bp)	-	PAS
**MYL6B**	ggccaccggaattaacccttcagggctgggggccgcgctatgccccgccccctccccagccccagacacggaccccgcaggagatgggtgcccccatccgcacactgtcctttggccaccggacatc (127bp)	gtgctgcagatccagtggggtccggacactgggccccgcaggcgaaagcacgttccagccaccaggaggccacctattgtttc**aaaataaagactgggttcctctcttggtttca** (115bp)	-	PAS

Table S6: Summary of selected human cellular UTRs. Sequences and features of selected UTRs. Sequences were obtained from UTR database website (http://utrdb.ba.itb.cnr.it/). A polyadenylation signal (PAS, bold) is located in every 3' UTR region of each of the cellular genes. CYBA UTR has furthermore an insulin 3'UTR stability element (INS_SCE, underlined) and the 3' UTR of DECR1 contains a small nucleotide polymorphism (SNP, big letters). In the 5' UTR region, DECR1 contains another SNP and the 5' UTR of GMFG includes an upstream open reading frame (uORF, cursive). In all the other UTRs no known 5' UTR regions could be identified.

Determination of GC content in 5' UTR

5' UTR	GC content (%)	Length (bases)
**CYBA**	72.2	36
**DECR1**	68.8	141
**GMFG**	57.3	110
**MAPBPIP**	65.9	164
**MYL6B**	70.1	127

Table S7: GC content of different cellular 5' UTRs and their lengths.

Primers for qRT-PCR

Oligo Name	DNA-Sequence 5'-3'
***Met*Luc_for pair 1**	gagacccaagctggctagcgt
***Met*Luc_rev pair 1**	tgcagaattctcatctgtcgccg
***Met*Luc_for pair 2**	gagacccaagctggctagcgt
***Met*Luc_rev pair 2**	tgcagaatcccggcttcgctg
**hBMP2_for**	agctcatttcaccaccggat
**hBMP2_rev**	acgtcgaagctctcccatct
**Human ß-Tubulin_for**	GAGGGCGAGGACGAGGCTTA
**Human ß-Tubulin_rev**	TCTAACAGAGGCAAAACTGAGCACC
**Human RunX2 v2_for**	TGCCTAGGCGCATTTCAGGTGC
**Human RunX2 v2_rev**	TGAGGTGACTGGCGGGGTGT
**Human OPN_for**	CTCCATTGACTCGAACGACTC
**Human OPN_rev**	CGTCTGTAGCATCAGGGTACTG

Table S8: Summary of primer pairs for full-length *Met*Luc, hBMP2 and osteogenic markers used for qRT-PCR measurements. DNA sequences are illustrated in 5’ to 3’ direction. Full length *Met*Luc without UTRs and mRNA with 5’ CYBA UTR shared primer pair 1. For full length MetLuc 5’+3’ UTRs primer pair 2 was used. Annealing temperature for all primers was 60°C.
